# Social Defeat Modulates T Helper Cell Percentages in Stress Susceptible and Resilient Mice

**DOI:** 10.3390/ijms20143512

**Published:** 2019-07-17

**Authors:** Oliver Ambrée, Christina Ruland, Peter Zwanzger, Luisa Klotz, Bernhard T Baune, Volker Arolt, Stefanie Scheu, Judith Alferink

**Affiliations:** 1 Department of Psychiatry, University of Münster, 48149 Münster, Germany; 2 Department of Behavioural Biology, University of Osnabrück, 49076 Osnabrück, Germany; 3 kbo-Inn-Salzach-Klinikum, 83512 Wasserburg am Inn, Germany; 4 Department of Psychiatry and Psychotherapy, Ludwig-Maximilians-Universität München, 80336 Munich, Germany; 5 Department of Neurology, University of Münster, 49149 Münster, Germany; 6 Department of Psychiatry, Melbourne Medical School, The University of Melbourne, Parkville, VIC 3010, Australia; 7 The Florey Institute of Neuroscience and Mental Health, The University of Melbourne, Parkville, VIC 3010, Australia; 8 Institute of Medical Microbiology and Hospital Hygiene, University of Düsseldorf, 40225 Düsseldorf, Germany; 9 Cluster of Excellence EXC 1003, Cells in Motion, University of Münster, 48149 Münster, Germany

**Keywords:** social defeat, Immune response, T cells, susceptibility, resilience, major depression, T_reg_ cells, Th17 cells, behavior, PPARγ

## Abstract

Altered adaptive immunity involving T lymphocytes has been found in depressed patients and in stress-induced depression-like behavior in animal models. Peripheral T cells play important roles in homeostasis and function of the central nervous system and thus modulate behavior. However, the T cell phenotype and function associated with susceptibility and resilience to depression remain largely unknown. Here, we characterized splenic T cells in susceptible and resilient mice after 10 days of social defeat stress (SDS). We found equally decreased T cell frequencies and comparably altered expression levels of genes associated with T helper (Th) cell function in resilient and susceptible mice. Interleukin (IL)-17 producing CD4^+^ and CD8^+^ T cell numbers in the spleen were significantly increased in susceptible mice. These animals further exhibited significantly reduced numbers of regulatory T cells (T_reg_) and decreased gene expression levels of TGF-β. Mice with enhanced Th17 differentiation induced by conditional deletion of PPARγ in CD4^+^ cells (CD4-PPARγ^KO^), an inhibitor of Th17 development, were equally susceptible to SDS when compared to CD4-PPARγ^WT^ controls. These data indicate that enhanced Th17 differentiation alone does not alter stress vulnerability. Thus, SDS promotes Th17 cell and suppresses T_reg_ cell differentiation predominantly in susceptible mice with yet unknown effects in immune responses after stress exposure.

## 1. Introduction

Stressful life events have been shown to result in long-term alterations of the immune system [1,2,3] and to increase the risk for major depressive disorder (MDD) [4,5]. Multiple studies have demonstrated a chronic mild inflammation characterized by increased levels of acute phase proteins, pro-inflammatory cytokines, and chemokines in depressed patients and stress-exposed individuals [6,7,8,9,10]. However, growing evidence also supports a role for the adaptive immune response and its cellular components, in particular T cells, in the pathophysiology of MDD [11,12] and in depression-like behaviors in rodents [13]. T cells have been shown to play an important role in neural plasticity and maintenance of CNS function [14,15,16]. Thus, alterations in the T cell compartment affect microglia function and adult neurogenesis that are involved in stress responses and MDD [17,18,19].

In patients with MDD and individuals exposed to stress, lower numbers of circulating T cells, as well as altered T cell responses, have been found by meta-analytic approaches [20,21]. Recent studies suggested that CD4^+^ T helper (Th)1, Th17 and T regulatory (T_reg_) cells are involved in the pathophysiology of MDD [13,22]. Interleukin-17 (IL-17)-producing Th17 cells exhibit potent inflammatory activity and have been functionally implicated in neuroinflammation and CNS autoimmunity [23]. On the other hand, T_reg_ cells play a key role in immune tolerance and downregulation of Th17 responses and exert inhibitory functions on immune effector cells and pro-inflammatory responses [24]. Individuals with MDD have been shown to exhibit altered percentages in circulating Th17 and T_reg_ cells. With regard to Th17 cells, different studies reported increased as well as decreased percentages of circulating Th17 cells in patients with MDD [25,26], while T_reg_ cells were mainly found to be decreased in the peripheral blood [26,27,28]. In summary, these findings point towards an imbalance of Th17 and T_reg_ cell populations in MDD.

Also in rodent models, stress-induced depression-like behavior has been shown to be associated with alterations of adaptive immune responses [29]. For example, various CD4^+^ Th cell subsets have been implicated in stress induced depression-like behavior: Percentages of Th17 cells were found to be elevated in brains of mice exhibiting learned helplessness and after chronic restraint stress [30]. Furthermore, adoptive transfer of Th17 cells increased depression-like behavior after foot shock stress while depletion of Th17 cells reduced the acquisition of learned helplessness [30]. However, the view that Th17 cells exclusively exhibit pathogenic actions has been challenged by studies pointing toward a potential beneficial role of Th17 cells in depression-like behavior and MDD. In a rodent model of depression-like behavior following chronic unpredictable mild stress, a decrease of Th17 cell percentages and an increase in percentages of T_reg_ cells was demonstrated [31]. In addition, Th17 cells have also been reported to promote adult hippocampal neurogenesis [16] which is usually associated with antidepressive effects [32,33]. A study in humans also pointed toward a potential beneficial role of Th17 cells in MDD by maintaining the functional and structural integrity of the brain [34]. Taken together, these findings in animals and humans suggest that circulating Th cells may contribute to stress responses and the development of MDD.

Recently, the nuclear receptor peroxisome proliferator-activated receptor gamma (PPARγ) has been identified as a key negative regulator of human and mouse Th17 differentiation and has been shown to suppress CNS autoimmunity [35,36]. In rodents, it has been demonstrated that PPARγ-agonists reduce depression-like behavior [37,38,39]. Furthermore, in MDD, PPARγ-agonists promoted enhanced remission [40,41]. However, whether PPARγ-mediated antidepressant effects are due to altered Th17 differentiation has not been investigated.

An important factor in understanding the consequences of stress on the organism, is a sound knowledge of the individual immune variations associated with stress vulnerability. It has been shown before that an early increase in plasma IL-6 levels predicts susceptibility to social defeat [42]. In addition, our earlier findings demonstrate that specific alterations in innate immune cells occur in monocytes and dendritic cells in susceptible mice that develop depression-like behavior after exposure to chronic social defeat [43]. However, the implication of T cells in stress susceptibility and resilience in this model remains undefined. In this study, we characterized T cell responses associated with stress vulnerability to social defeat by assessing expression levels of T cell differentiation and effector genes and numbers of cytokine-producing T cells in socially defeated animals. In addition, we examined the effect of increased IL-17 producing CD4^+^ T cells on stress vulnerability in socially defeated CD4^+^ T cell-specific PPARγ knockout mice. Our data identified a specific pattern of T cell responses associated with social defeat stress and point toward an involvement of the adaptive immune system as cellular contributor to brain homeostasis relevant for MDD and the physiological stress response.

## 2. Results

### 2.1. Susceptible Mice Show Social Avoidance after Social Defeat Stress

**Figure 1:**
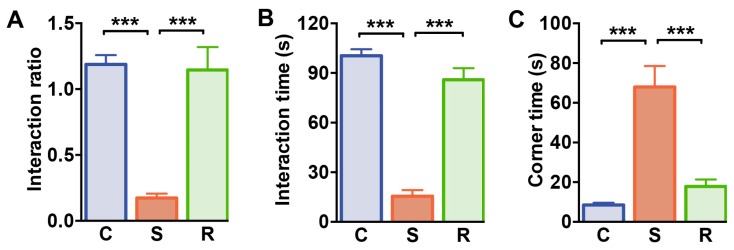
Social interaction test. (**A**) Interaction ratio, (**B**) the time spent in the interaction zone during the social interaction trial, and (**C**) the time spent in the corners on the opposite site of the interaction enclosure. Bar graphs represent mean + SEM. C: control, S: susceptible, R: resilient. *n_C_* = 15, *n_S_* = 16, n_R_ = 11. ***: *p* < 0.001 (Bonferroni post hoc).

To study T cell responses associated with susceptibility and resilience to prolonged stress, we utilized repeated social defeat in mice as a paradigm for social stress [43,44]. For this, C57BL/6J mice were subjected to repeated social defeats over 10 days. After ten days of exposure to dominant conspecifics and repeated social defeat, we assessed social interaction behavior to determine susceptible and resilient individuals. In analogy to our previous study [43], susceptible mice had a significantly lower interaction ratio than control and resilient animals, whose interaction ratio was comparable to that of control animals (Figure 1A, C vs S: *p* < 0.001; S vs. R: *p* < 0.001, see Table S1 for details of statistics). In addition, the behavior of control and resilient mice clearly differed with regard to the time spent in the interaction zone that was reduced in susceptible mice (Figure 1B, C vs S: *p* < 0.001; S vs. R: *p* < 0.001). In line with that, the time spent in the corner zone was increased in susceptible animals when compared to resilient animals and undefeated controls (Figure 1C, C vs S: *p* < 0.001; S vs. R: *p* < 0.001).

### 2.2. Expression Levels of Molecules Associated with T Cell Differentiation and Function Were Reduced after Social Defeat

**Figure 2:**
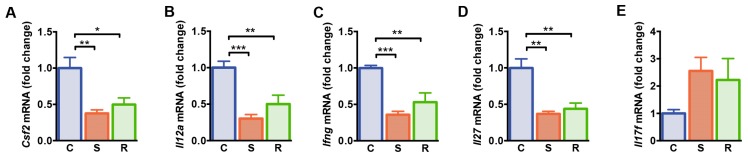
Gene expression analysis of splenocytes from control, susceptible and resilient mice after 10 days of social defeat. (**A**) mRNA expression of *Csf2*, (**B**) *Il12a*, (**C**) *Ifng*, (**D**) *Il27*, and (**E**) *Il17f*. Expression levels were normalized to the mean expression of housekeeping genes *Gapdh* and *Hsp90ab1.* Fold changes were calculated relative to control mice. Bar graphs represent mean + SEM. C: control, S: susceptible, R: resilient. *n_C_* = 6, *n_S_* = 6, *n_R_* = 4. *: *p* < 0.05, **: *p* < 0.01, ***: *p* < 0.001 (Bonferroni post hoc).

We next determined expression of genes associated with T cell differentiation and function in the spleen of resilient and susceptible mice after social defeat and controls. A panel of genes that were differentially expressed after social defeat, was selected based on the results of a qPCR-based gene array on 84 genes encoding pro- and anti-inflammatory cytokines and chemokines (Table S2). Granulocyte-macrophage colony-stimulating factor (GM-CSF; also designated as colony stimulating factor 2, CSF2) represents a pro-inflammatory mediator for T cell function and myeloid cell responses during tissue inflammation [45,46]. We found lower levels of *Csf2* mRNA in socially defeated mice when compared to non-defeated controls, independent of the susceptible or resilient phenotype of defeated animals (Figure 2A, C vs. S: *p* = 0.003, C vs. R: *p* = 0.026). The expression levels of genes encoding the Th1 differentiation cytokine interleukin (IL)-12 and interferon (IFN)-γ were reduced in the spleen of susceptible and resilient mice after social defeat when compared to non-defeated controls (Figure 2B,C, *Il12a*: C vs. S: *p* < 0.001, C vs. R: *p* = 0.006; *Ifng*: C vs. S: *p* < 0.001, C vs. R: *p* = 0.001). In addition, mRNA levels of *Il27*, the gene encoding the pleiotropic cytokine IL-27, an inhibitor of Th17 development [47], were reduced in susceptible and resilient mice compared to controls (Figure 2D, C vs. S: *p* = 0.001, C vs. R: *p* = 0.003). In accordance, expression of *Il17f* mRNA encoding the pro-inflammatory cytokine IL-17F tended to be increased in defeated mice (Figure 2E, *p* = 0.067). Thus, expression levels of genes associated with Th cell functions are modulated after social defeat stress similarly in susceptible and resilient animals. 

### 2.3. Reduced Percentages of T Lymphocytes in Susceptible and Resilient Mice after Social Defeat Stress

**Figure 3:**
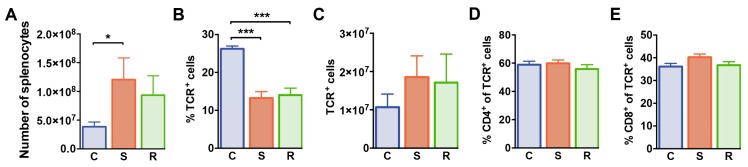
Numbers of splenocytes and αβ T cells in mice after social defeat and control animals. (**A**) Absolute numbers of splenocytes in control, susceptible and resilient mice. (**B**) Percentages and (**C**) numbers of TCR^+^ T cells, (**D**) percentages of CD4^+^ cells among TCR^+^ T cells, and (**E**) percentages of CD8^+^ cells among TCR^+^ T cells in the spleen as determined by flow cytometry. Bar graphs represent mean + SEM. C: control, S: susceptible, R: resilient. *n_C_* = 10, *n_S_* = 11, *n_R_* = 9. *: *p* < 0.05, ***: *p* < 0.001 (Bonferroni post hoc).

We next examined splenocytes and T cell subsets in the spleen of these animals by flow cytometry. After 10 days of social defeat, the numbers of splenic mononuclear cells were increased in susceptible mice when compared to control animals (Figure 3A, *p* = 0.019). Percentages of splenic αβ T cells were markedly reduced in defeated mice when compared to non-defeated controls (Figure 3B) resulting in equivalent αβ T cell numbers in the spleen of these animals (Figure 3C). Frequencies of CD4^+^ and CD8^+^ cells among αβ T cells and absolute numbers of these subsets were comparable in all groups excluding that social defeat differentially affected homeostasis of these T cell subsets (Figure 3D,E, CD4 %: *p* = 0.551; #: *p* = 0.092; CD8 %: *p* = 0.099; #: *p* = 0.042). Thus, social defeat stress reduces percentages of T cells in the spleen independent of the behavioral outcome with regard to susceptibility or resilience. 

### 2.4. Increased Numbers of IL-17 Producing T Cells after Social Defeat Stress

**Figure 4:**
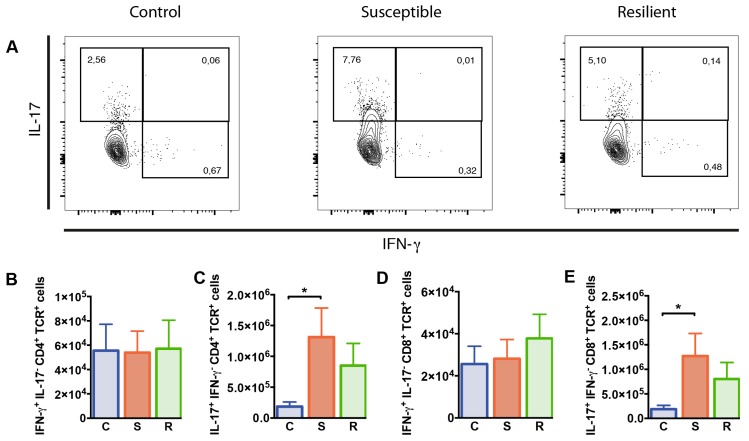
Cytokine expression by CD4^+^ and CD8^+^ T cells from the spleen of mice after social defeat. Representative contour plots showing expression of (**A**) IFN-γ and IL-17 in CD4^+^ T cells from the spleen as determined by flow cytometry. (**B**) Numbers of IFN-γ and (**C**) IL-17 producing CD4^+^ T cells. (**D**) Numbers of IFN-γ cells and (**E**) IL-17 producing CD8^+^ T cells. Bar graphs represent mean + SEM. C: control, S: susceptible, R: resilient. *n_C_* = 9, *n_S_* = 10, *n_R_* = 9. *: *p* < 0.05 (Bonferroni post hoc).

To study whether T cell functions were differentially affected in susceptible versus resilient mice following social defeat, we studied the cytokine producing capacity of T cells in these animals. For this, interferon (IFN)-γ and IL-17 production by splenic CD4^+^ and CD8^+^ T cells was determined after 10 days of social defeat by flow cytometry. Percentages of CD4^+^ T cells producing IFN-γ but not IL-17 were decreased in susceptible mice after social defeat when compared to controls (*p* = 0.019); however, absolute numbers were comparable between all groups (Figure 4B). In contrast, IL-17^+^ IFN-γ^−^ CD4^+^ T cell proportions and numbers were elevated in susceptible animals after social defeat (Figure 4C, %: *p* = 0.006; #: *p* = 0.032). Percentages and absolute numbers of CD4^+^ T cells co-expressing IFN-γ and IL-17 were rather low and similarly distributed in all three groups. The entire population of IL-17 producing CD4^+^ T cells, comprising IL-17^+^ IFN-γ^+^ and IL-17^+^ IFN-γ^−^ cells, showed similar effects as the IL-17^+^ IFN-γ^−^ population. Susceptible mice again presented increased proportions and numbers compared to controls (%: *p* = 0.002; #: *p* = 0.015). Percentages and numbers of CD8^+^ T cells producing IFN-γ did not differ between the groups (Table S1, Figure 4D). However, we found enhanced percentages and absolute numbers of IL-17 expressing CD8^+^ T cells in the spleen of susceptible mice after social defeat compared to controls (Table S1, %: *p* = 0.006; #: Figure 4E, *p* = 0.036). Thus, social defeat affects CD4^+^ and CD8^+^ T cells producing IL-17 in susceptible animals. 

### 2.5. Reduced Numbers of Regulatory T Cells after Social Defeat

**Figure 5:**
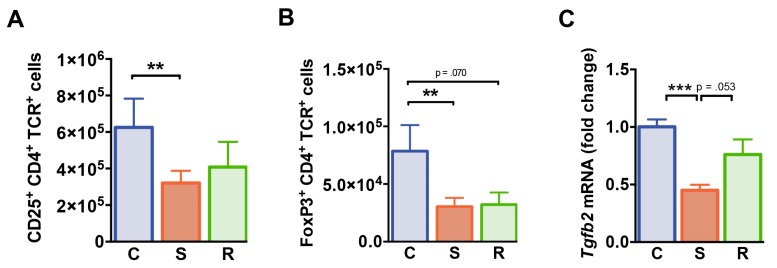
T regulatory cells. (**A**) Numbers of CD25^+^ CD4^+^ T cells. (**B**) Absolute numbers of FoxP3^+^ CD4^+^ T cells. (**C**) mRNA expression of *Tgfb2* in splenocytes. Expression levels were normalized to the mean expression of housekeeping genes *Gapdh* and *Hsp90ab1.* Fold changes were calculated relative to control mice. Bar graphs represent mean + SEM. C: control, S: susceptible, R: resilient. *n_C_* = 15, *n_S_* = 16, *n_R_* = 11. **: *p* < 0.01, ***: *p* < 0.001 (Bonferroni post hoc).

We next investigated whether distinct behavioral changes after social defeat affect the immunoregulatory T cell compartment. While percentages of CD4^+^ T cells expressing the IL-2 receptor α-chain (CD25) were equivalent in all groups of mice (Table S1), absolute numbers of CD25^+^ CD4^+^ T cells were significantly reduced in susceptible animals (Figure 5A, *p* = 0.003). FoxP3 expressing CD4^+^ T cells specifically linked to immune regulation were markedly reduced in numbers in susceptible mice when compared to controls (Figure 5B, *p* = 0.009). In resilient animals, a similar trend was observed (*p* = 0.070). Splenic FoxP3^+^ T_reg_ cell percentages were not affected by social defeat (Table S1). Furthermore, splenic mRNA levels of transforming growth factor β (*Tgfb*) encoding the immunomodulatory TGF β which is crucial for T_reg_ cell-mediated suppression in vivo were significantly reduced in susceptible mice compared to control animals (Figure 5C, C vs. S: *p* < 0.001, S vs. R: *p* = 0.053). Together these findings point toward an altered immunoregulatory status in defeated mice.

### 2.6. Enhancement of Th17 Differentiation Did Not Alter Behavioral Responses to Social Defeat

**Figure 6:**
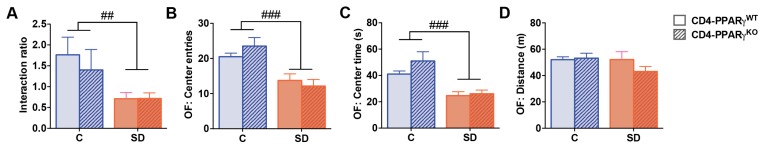
Behavioral data of CD4-specific PPARγ knockout (CD4-PPARγ^KO^) mice and Cre-negative floxed controls (CD4-PPARγ^WT^) after 10 days of social defeat. (**A**) The interaction ratio of the social interaction test. (**B**) The number of center entries, (**C**) the time spent in the center, and (**D**) the distance traveled in the open-field test. Data represent mean + SEM. CD4-PPARγ^WT^ Control (C): *n* = 6, CD4-PPARγ^WT^ social defeat (SD): *n* = 11, CD4-PPARγ^KO^ Control: *n* = 6, CD4-PPARγ^KO^ SD: *n* = 8, ##: main effect of stress, *p* < 0.01, ###: *p* < 0.001.

Finally, we investigated whether the increase in IL-17 producing CD4^+^ T cell percentages observed in defeated mice is sufficient to alter behavioral responses to social defeat stress. We therefore utilized mice with CD4-specific knockout of PPARγ, a key negative regulator of Th17 differentiation [36]. In CD4-PPARγ^KO^ mice, Th17 differentiation is strongly increased, while Th1, Th2, or T_reg_ cell differentiation is not affected [36]. We subjected CD4-PPARγ^KO^ mice and CD4-PPARγ^WT^ controls to social defeat and analyzed social as well as anxiety-related behavior. Socially defeated CD4-PPARγ^KO^ mice showed an equivalently reduced interaction ratio in the social interaction test compared to CD4-PPARγ^WT^ controls (Figure 6A, main effect of stress: *p* = 0.005). In the open-field test, CD4-PPARγ^KO^ mice also showed comparably reduced center entries and time spent in the center when compared to CD4-PPARγ^WT^ controls after ten days of social defeat (Figure 6B,C; Center entries: main effect of stress: *p* < 0.001; Center time: main effect of stress: *p* < 0.001). These data indicate similar anxiety-related behavior in both genotypes that were equally affected by stress exposure. The distance traveled in the open field test was not affected by social defeat or genotype (Figure 6D). These findings suggest that PPARγ−mediated changes in T cell differentiation and function do not modulate social and anxiety-like behavior, neither under control conditions nor after ten days of social defeat. We also analyzed these behaviors subdividing the defeated groups into susceptible and resilient mice. Again, no effects of genotype or interaction effects of genotype and stress exposure could be detected (Figure S1) suggesting that an enhanced Th17 differentiation status induced by CD4-specific deficiency of PPARγ is not sufficient to alter emotional behavior or stress vulnerability.

## 3. Discussion

Stress is known to evoke long-term alterations of the adaptive immune response [1,2,3]. At the same time, it is well established that individuals exhibit considerable variability in behavioral responses to stressors, and even genetically identical inbred mouse strains show an individual variability in the sensitivity to social stressors [48]. How specific adaptive immune alterations are correlated with individual stress susceptibility and resilience is poorly characterized. A better understanding of the underlying mechanisms is of major importance due to the enormous health burden of stress-related affective disorders. 

In this study, we characterized alterations of the adaptive immune response associated with stress susceptibility and resilience in a mouse model of social defeat. Our data show reduced T cell percentages and altered expression levels of T cell differentiation and effector genes in the spleen of mice exposed to social defeat irrespective of a stress susceptible or resilient behavioral phenotype. We further observed greatly increased numbers of splenic IL-17 producing CD4^+^ and CD8^+^ T cells in susceptible animals compared to controls while regulatory T cells were reduced after social defeat. 

A decrease in T cell numbers or percentages has been reported before in various animal models of acute and chronic stress [49,50,51,52]. Stress exposure results in an enhanced release of glucocorticoids and catecholamines that may induce apoptosis in peripheral T cells [53,54]. Enhanced T cell apoptosis following stressful events has further been attributed to lack of tryptophan, an essential factor in T cell proliferation [55]. In addition, inputs from adrenergic nerves have been shown to affect T cell trafficking, since stimulation of β2-adrenergic receptors on T cells reduces their egress from lymph nodes [56]. Furthermore, T cells from MDD patients showed lower expression of the chemokine receptors CXCR3 and CCR6 that modulate T cell differentiation and trafficking [57]. Of note, it has been suggested that T cells exert stress protective effects based on observations that T cell deficient BALB/c nude mice were more vulnerable to brief exposures to foot shocks than T cell competent mice on the same genetic background [58]. Our findings of reduced percentages but not numbers of splenic T cells point toward an altered cellular composition in the spleen after social defeat. It has frequently been shown that stress results in an increase in the numbers of innate immune cells including natural killer cells, neutrophils, and monocytes [49,59]. In particular, our earlier findings demonstrating that mice after chronic social defeat show higher numbers of splenic myeloid cells [43] may explain the here observed higher cellularity of the spleen.

In the present study, IL-17-producing CD4^+^ T cells, classified before as pathogenic Th17 cells in inflammatory responses and neuroinflammation, were markedly enhanced in susceptible mice in response to social defeat stress. Accordingly, levels of the gene encoding IL-27, a cytokine mediating suppressive effects on the Th17 lineage, were lower in the spleen of these animals [47,60]. These findings are in line with previous studies demonstrating that the vulnerability for the development of learned helplessness was dependent on increased Th17 responses [30]. It has also been shown that the cytokine IL-6, which is required for the induction of Th17 differentiation [24,61], is indicative of stress susceptibility [42]. However, in CD4-specific PPARγ deficient animals exhibiting enhanced Th17 differentiation [36], social defeat stress had the same effect on the behavioral level when compared to PPARγ-competent controls. It is important to note that the underlying mechanism for increased Th17 differentiation induced by CD4-specific deficiency of PPARγ might differ from the mechanism responsible for the stress-induced Th17 shift in our model. Further research will focus on defining potential regulators involved in Th17 differentiation after social defeat, e.g., the signal transducer and activator of transcription 3 (STAT3) and the transcription factors IFN regulatory factor 4 (IRF4), c-Rel and RelA/p65 required for Th17 differentiation and responses. In addition, analyses of the Th1-specific T-box transcription factor T-bet and the Th17 specific RAR-related orphan receptor gamma t (RORγt) will provide better insights into the differentiation of T helper cells in this model.

Our findings suggest that CD4^+^ cell-specific deletion of PPARγ may not be sufficient to promote stress resilience in this model. In contrast, an anxiolytic effect of neuronal deletion of PPARγ on the emotional response to acute stress has been described before [62]. It is, therefore, likely that PPARγ operates on various cellular levels in modulating anxiety- and depressive-like behavior. It is still unclear, however, whether alterations in the here-studied T cell subsets are the cause or consequence of stress susceptibility and whether IL-17 producing T cells have differential implications in stress susceptible and resilient mice.

T_reg_ cells are predominantly viewed as mediators for immune tolerance and suppression [24]. We observed reduced T_reg_ numbers in both, susceptible and to a minor extent also in resilient socially defeated mice. In analogy, reduced T_reg_ numbers associated with systemic T cell activation have been found during subordinate colony housing [63], suggesting that a reduction in these cells might contribute to an overall pro-inflammatory state in these animals. In the same model, T_reg_ cells are necessary to induce stress resilience by immunization with *Mycobacterium vaccae* [64] suggesting a functional role of these cells in mediating stress vulnerability and resilience. However, also controversial findings on T_reg_ cells in murine stress models have been reported and may be explained by the diversity of models and the different time points studied. For example, T_reg_ cell proportions have been found increased in mice due to chronic unpredictable mild stress [31] and enhanced frequencies of peripheral T_reg_ cells and an elevated suppressive function of these cells have further been found after chronic immobilization of mice [65]. In a model of learned helplessness induced by mild inescapable foot shocks, no difference was found in percentages of T_reg_ cells between controls and mice exhibiting learned helplessness [30]. Future studies will therefore have to focus on the impact of T_reg_ cells in this model to better understand the role of these cells in the regulation of emotional behavior.

In the spleen of resilient mice, we observed an “intermediate” immune pattern characterized by lower numbers and percentages of splenic IL-17 secreting T cells when compared to susceptible mice but higher values in these categories than controls, albeit those values did not reach significance. Thus, our analysis of the adaptive immune status after social defeat did not reveal overt differences in susceptible and resilient animals in contrast to our earlier findings regarding the innate immune system. Herein we demonstrated specific alterations in susceptible mice among those an enhanced maturation of dendritic cells in the spleen, and increased brain immigration of CCR2^+^ Ly6C^hi^ monocytes representing an inflammatory phenotype [43].

In conclusion, our study provides evidence that specific alterations of the adaptive immune responses, which are involved in maintaining brain function, plasticity and behavior, are induced by social defeat stress. Future studies in this model may close the knowledge gap concerning the link between adaptive immune responses and stress vulnerability by analyzing the impact of T_reg_ cells and TGF-β on pro-inflammatory responses, kynurenine metabolism, and microglial activation. In addition, longitudinal immune studies in rodents exposed to chronic stress and humans during clinical course of MDD are necessary to yield a better understanding of the pathophysiology of affective disorders.

## 4. Materials and Methods 

### 4.1. Mice and Housing Conditions

Five-week-old, male C57BL/6J mice were purchased at Charles River (Sulzfeld, Germany). After a habituation period of two weeks, the social defeat experiments started. CD-1 mice from our in-house breeding facility were used as resident animals for the social defeat paradigm and social interaction partners. These mice were older than 3 months and most of them had mating experience. Their level of aggressive behavior was tested before chosen for the experiment (latency to attack intruder should be less than 30 s). All animals were housed at 22 ± 2 °C and humidity of 55 ± 10% under a 12 h:12 h light-dark cycle, with lights on at 6 am. Food and water were available ad libitum. This study was performed in accordance with the regulations covering animal experimentation in Germany and the EU (European Communities Council Directive 2010/63/EU). The project was approved by the local authority and the Animal Welfare Officer of the University of Münster (84-02.04.2013.A320, 31 October 2013). All efforts were made in order to minimize animal suffering and reduce the number of animals used.

### 4.2. Social Defeat Paradigm

The social defeat paradigm was performed as described before [43]. Briefly, experimental mice were inserted into the cage of an aggressive, older and heavier CD-1 mouse for 10 min per day. After 10 min direct physical contact, animals were separated by a perforated Plexiglas wall and kept on opposite sides of the same cage for 24 h to maintain visual and olfactory contact. This procedure was repeated daily with another CD-1 mouse. After the final confrontation on day 10, experimental mice were housed singly in Makrolon type II cages. Control mice were housed in the same type of cage as experimental mice. The degree of agonistic interactions was observed by an experienced observer who terminated the sessions and separated the animals immediately in case that escalated fighting occurred before 10 min passed [43].

### 4.3. Social Interaction Test

One day after the last social defeat session, the social interaction test was conducted as described before [43,66]. Briefly, it comprised two trials of 150 s each, one with an empty enclosure, the second with an unfamiliar CD-1 mouse therein. The time spent in the interaction zone, defined as the area surrounding the exploration enclosure 8 cm to each side was recorded in both trials by ANY-maze tracking software (Stoelting, Dublin, Ireland). An interaction ratio was calculated as time spent in the interaction zone during the second trial with mouse divided by the time spent in the zone during the first trial with the empty enclosure. When the interaction ratio was less than 0.5, animals were defined as susceptible, otherwise as resilient [43].

### 4.4. Open Field Test

The open field test was conducted as described before [67]. Briefly, mice were introduced into one corner of an 80 cm × 80 cm wooden box with 40 cm high walls and allowed to freely explore the box for 10 min. The distance traveled, the number of entries into the 40 cm × 40 cm center area and the time spent therein were automatically recorded by ANY-maze tracking software (Stoelting, Dublin, Ireland).

### 4.5. Gene Expression Analysis

The day after the social interaction test, spleens of control (*n* = 6), susceptible (*n* = 6) and resilient (*n* = 4) mice were dissected and immediately snap-frozen in liquid nitrogen. RNA was extracted from half spleens using the RNeasy Midi kit (Qiagen, Hilden, Germany). RNA was reverse transcribed to generate cDNA using the RT^2^ HT First Strand kit (Qiagen). RT2 Profiler PCR Arrays for mouse cytokines and chemokines were run for control (*n* = 3), susceptible (*n* = 3) and resilient mice (*n* = 2) according to the manufacturer’s instructions on an ABI 7900 HT PCR system (Life Technologies, Darmstadt, Germany). Based on the results of this array, candidate genes were chosen and Taqman assays were run in triplicates as described before [68]. The following candidate genes were assessed using inventoried assays (Life Technologies, Darmstadt, Germany): *Csf2* (Mm01290062_m1), *Il12a* (Mm00434165_m1), *Ifng* (Mm01168134_m1), *Il27* (Mm00461162_m1), *Il17f* (Mm00521423_m1), *Tgfb2* (Mm00436955_m1). To calculate ΔCt levels for each of the candidate genes, average Cts of housekeeping genes *Hsp90ab1* (Mm00833431_g1) and *Gapdh* (Mm99999915_g1) were used. Fold changes were calculated as 2^–ΔΔCt^ using the non-defeated control group as reference.

### 4.6. Flow Cytometry

Spleens of control (*n* = 15), susceptible (*n* = 16) and resilient (*n* = 11) mice were homogenized after transcardial perfusion and a single cell suspension was received as described before [43]. Due to limitations in the number of animals that could be dissected on a single day, the group of animals was divided into three cohorts as shown in Table S3.

The following antibodies (purchased at Biolegend, San Diego, CA, USA) were used for fluorescent staining of splenocytes: FITC or PerCP-Cy5.5-conjugated anti-mouse TCR β-chain (clone H57-597), APC-Cy7-conjugated anti-mouse CD4 (clone RM4-5), APC or PE-Cy7-conjugated anti-mouse CD8a (clone 53-6.7), BV510-conjugated anti-mouse IFN-γ (clone XMG1.2), PE-conjugated anti-mouse IL-17A (clone eBio17B7), PE-Cy7-conjugated anti-mouse CD25 (clone PC-61) and PE-conjugated anti-mouse FoxP3 (clone FJK-16s).

Intracellular staining was performed according to the manufacturer’s instructions using the Fixation/Permeabilization kit (BD Cytofix/Cytoperm), intranuclear staining using the FOXP3 Fix/Perm buffer set (Biolegend, London, UK). For ex vivo stimulation of lymphocytes, 5 × 10^6^ splenocytes were incubated with PMA (10 ng/mL) and ionomycin (500 ng/mL) plus Monensin and Brefeldin A (Biolegend, London, UK) for 10 h overnight. Samples were acquired on a FACSCanto II (BD Biosciences, East Rutherford, NJ) flow cytometer and analyzed by FlowJo v10. The gating strategy comprised life gates (SSC-A vs- FSC-A) to exclude debris and dead cells. Subsequently, doublets were gated out by comparing sideward and forward scatter height and width (SSC-H vs SSC-W and FSC-H vs. FSC-W). TCR^+^ cells were considered as T cells, CD4^+^ and CD8^+^ T cells were determined on TCR^+^ pregates. IFN-γ^+^, IL-17^+^ cells were determined on CD4^+^ or CD8^+^ T cell pregates, respectively. Accordingly, CD25^+^ and FoxP3^+^ Th cells were assessed on CD4^+^ T cell pregates.

### 4.7. Statistics

Data obtained in independent cohorts were combined and analyzed by analysis of covariance (ANCOVA) with stress phenotype as fixed factor and cohort as covariate. In case of significant effects of the stress phenotype, Bonferroni post hoc tests were calculated. The null-hypothesis was rejected for *p* < 0.05. All analyzes were calculated with SPSS 24 (IBM). 

## Supplementary Materials

Supplementary materials can be found at https://www.mdpi.com/1422-0067/20/14/3512/s1.

## Abbreviations

CD	Cluster of differentiation
IFN	Interferone
IL	Interleukin
MDD	Major depressive disorder
PPAR-γ	peroxisome proliferator-activated receptor γ
TGF-β	Transforming growth factor-β
Th cell	T helper cell
T_reg_ cell	T regulatory cell
