# Supplementary material for: Social Defeat Modulates T Helper Cell Percentages in Stress Susceptible and Resilient Mice
Source: Int J Mol Sci. 2019 Jul 17;20(14):3512. doi: 10.3390/ijms20143512 (PMC6678569; doi:10.3390/ijms20143512)
Supplement: Supplementary file 1 [file ijms-20-03512-s001.zip › Supplementary_Figure_1.pdf]

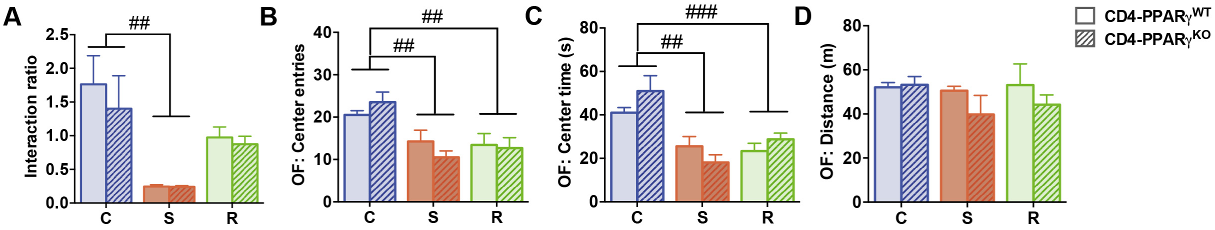

Figure S1. Behavioral data of CD4-specific PPARγ knockout (CD4-PPARγ^{KO}) mice and Cre-negative floxed controls (CD4-PPARγ^{WT}) after 10 days of social defeat subdivided into stress susceptible and resilient mice. (A) In both genotypes, the interaction ratio of the social interaction test was significantly reduced in susceptible mice compared to controls. (B) The number of center entries in the open-field as well as (C) the time spent in the center was reduced in both defeated groups compared to controls independent of the genotype. (D) The distance traveled in the open-field test was neither affected by social defeat stress nor genotype. Data represent mean + SEM. CD4-PPARγ^{WT} Control (C): n = 6, susceptible (S): n = 4, resilient (R): n = 7. CD4-PPARγ^{KO} Control: n = 6, CD4-PPARγ^{KO} S: n = 2, R: n = 6. Statistics: Bonferroni posthoc comparisons for main effects of stress condition, ##: $p < 0.01$, ###: $p < 0.001$.
