# Supplementary material for: Social Defeat Modulates T Helper Cell Percentages in Stress Susceptible and Resilient Mice
Source: Int J Mol Sci. 2019 Jul 17;20(14):3512. doi: 10.3390/ijms20143512 (PMC6678569; doi:10.3390/ijms20143512)
Supplement: Supplementary file 1 [file ijms-20-03512-s001.zip › Supplementary_Table_1.pdf]

Table S1. Overview of the statistical data for all analyzed parameters in socially defeated C57BL/6J mice. C: control, S: susceptible, R: resilient.

Parameter	mean ± SEM			ANCOVA		Bonferroni posthoc		
	C	S	R			C vs. S	C vs. R	S vs. R
Social interaction test								
n	15	16	11					
Interaction ratio	1.19 ± 0.07	0.17 ± 0.03	1.14 ± 0.17	$F(2,39) = 41.70$	**$p < 0.001$**	**$p < 0.001$**	$p > 0.999$	**$p < 0.001$**
Interaction time (s)	100.43 ± 3.95	15.53 ± 3.72	85.85 ± 7.03	$F(2,39) = 101.67$	**$p < 0.001$**	**$p < 0.001$**	$p = 0.129$	**$p < 0.001$**
Corner time (s)	8.58 ± 1.03	67.93 ± 10.62	17.87 ± 3.50	$F(2,39) = 21.09$	**$p < 0.001$**	**$p < 0.001$**	$p > 0.999$	**$p < 0.001$**
Taqman gene expression analysis								
n	6	6	4					
Csf2	1.00 ± 0.15	0.37 ± 0.05	0.50 ± 0.09	$F(2,13) = 10.10$	**$p = 0.002$**	**$p = 0.003$**	**$p = 0.026$**	$p > 0.999$
Il12a	1.00 ± 0.09	0.30 ± 0.06	0.50 ± 0.12	$F(2,13) = 19.51$	**$p < 0.001$**	**$p < 0.001$**	**$p = 0.006$**	$p = 0.424$
Ifng	1.00 ± 0.03	0.36 ± 0.04	0.53 ± 0.13	$F(2,13) = 29.10$	**$p < 0.001$**	**$p < 0.001$**	**$p = 0.001$**	$p = 0.307$
Il27	1.00 ± 0.12	0.37 ± 0.04	0.44 ± 0.08	$F(2,13) = 15.57$	**$p < 0.001$**	**$p = 0.001$**	**$p = 0.003$**	$p > 0.999$
Il17f	1.00 ± 0.14	2.56 ± 0.49	2.22 ± 0.78	$F(2,13) = 3.36$	$p = 0.067$			
Tgfb2	1.00 ± 0.06	0.45 ± 0.05	0.76 ± 0.13	$F(2,13) = 14.78$	**$p < 0.001$**	**$p < 0.001$**	$p = 0.155$	$p = 0.053$
n	15	16	11					
Absolute spleen cell number ($x10^7$)	3.85 ± 0.82	12.06 ± 3.79	9.36 ± 3.37	$F(2,37) = 4.40$	**$p = 0.019$**	**$p = 0.019$**	$p = 0.182$	$p > 0.999$
Spleen cells unstimulated								
n	10	11	9					
% TCR^+	26.24 ± 0.72	13.25 ± 1.67	14.02 ± 1.84	$F(2,26) = 27.11$	**$p < 0.001$**	**$p < 0.001$**	**$p < 0.001$**	$p > 0.999$
# TCR^+ ($x 10^7$)	1.07 ± 0.34	1.86 ± 0.56	1.71 ± 0.74	$F(2,26) = 2.74$	$p = 0.083$			
% $CD4^+$ (TCR^+ pregate)	58.88 ± 2.46	59.90 ± 2.33	55.86 ± 3.10	$F(2,26) = 0.61$	$p = 0.551$			
# $CD4^+$ TCR^+ ($x 10^7$)	0.67 ± 0.22	1.22 ± 0.38	1.06 ± 0.46	$F(2,26) = 2.61$	$p = 0.092$			
% $CD8^+$ (TCR^+ pregate)	36.16 ± 1.36	40.27 ± 1.35	36.79 ± 1.49	$F(2,26) = 2.54$	$p = 0.099$			
# $CD8^+$ TCR^+ ($x10^6$)	3.66 ± 1.16	7.70 ± 2.35	6.23 ± 2.69	$F(2,26) = 3.59$	**$p = 0.042$**	$p = 0.062$	$p = 0.128$	$p > 0.999$

Table S1 continued

Parameter	mean ± SEM			ANCOVA		Bonferroni posthoc		
	C	S	R			C vs. S	C vs. R	S vs. R
PMA/Iono stimulated spleen cells								
n	9	11	9					
% IFN-γ^+ IL-17A$^-$ (CD4$^+$ TCR$^+$ pregate)	1.49 ± 0.27	0.71 ± 0.13	1.30 ± 0.30	F(2,25) = 4.82	**p = 0.017**	**p = 0.019**	p > 0.999	p = 0.129
# IFN-γ^+ IL-17A$^-$ CD4$^+$ TCR$^+$ (x 10^4)	5.55 ± 2.17	5.40 ± 1.77	5.71 ± 2.35	F(2,25) = 0.69	p = 0.511			
% IL-17A$^+$ IFN-γ^- (CD4$^+$ TCR$^+$ pregate)	1.30 ± 0.50	5.73 ± 1.78	3.57 ± 1.45	F(2,25) = 6.05	**p = 0.007**	**p = 0.006**	p = 0.157	p = 0.578
# IL-17A$^+$ IFN-γ^- CD4$^+$ TCR$^+$ (x 10^6)	0.19 ± 0.08	1.31 ± 0.47	0.85 ± 0.36	F(2,25) = 3.89	**p = 0.034**	**p = 0.032**	p = 0.242	p > 0.999
% IFN-γ^+ IL-17A$^+$ (CD4$^+$ TCR$^+$ pregate)	0.03 ± 0.01	0.01 ± 0.01	0.04 ± 0.02	F(2,25) = 1.78	p = 0.189			
# IFN-γ^+ IL-17A$^+$ CD4$^+$ TCR$^+$ (x 10^3)	3.32 ± 1.54	2.51 ± 1.51	10.16 ± 6.95	F(2,25) = 1.68	p = 0.207			
% IL-17A$^+$ IFN-$\gamma^{+/-}$ (CD4$^+$ TCR$^+$ pregate)	1.32 ± 0.51	5.74 ± 1.78	3.61 ± 1.46	F(2,25) = 7.58	**p = 0.003**	**p = 0.002**	p = 0.075	p = 0.603
# IL-17A$^+$ IFN-$\gamma^{+/-}$ CD4$^+$ TCR$^+$ (x 10^6)	0.19 ± 0.08	1.32 ± 0.47	0.86 ± 0.36	F(2,25) = 4.90	**p = 0.016**	**p = 0.015**	p = 0.141	p > 0.999
% IFN-γ^+ IL-17A$^-$ (CD8$^+$ TCR$^+$ pregate)	2.19 ± 0.72	1.27 ± 0.46	3.36 ± 1.11	F(2,25) = 2.95	p = 0.071			
# IFN-γ^+ IL-17A$^-$ CD8$^+$ TCR$^+$ (x 10^4)	2.56 ± 0.85	2.82 ± 0.91	3.78 ± 1.14	F(2,25) = 1.68	p = 0.206			
% IL-17A$^+$ (CD8$^+$ TCR$^+$ pregate)	1.93 ± 0.75	6.61 ± 2.00	4.56 ± 1.77	F(2,25) = 6.01	**p = 0.007**	**p = 0.006**	p = 0.097	p = 0.911
# IL-17A$^+$ CD8$^+$ TCR$^+$ (x 10^6)	0.19 ± 0.08	1.27 ± 0.46	0.80 ± 0.34	F(2,25) = 3.75	**p = 0.038**	**p = 0.036**	p = 0.289	p > 0.999
% IFN-γ^+ IL-17A$^+$ (CD8$^+$ TCR$^+$ pregate)	0.02 ± 0.01	0.01 ± 0.004	0.03 ± 0.01	F(2,25) = 1.93	p = 0.166			
# IFN-γ^+ IL-17A$^+$ CD8$^+$ TCR$^+$ (x 10^3)	1.09 ± 0.73	1.05 ± 0.68	3.63 ± 2.39	F(2,25) = 1.58	p = 0.227			
Intranuclear FoxP3 stained spleen cells								
n	15	16	11					
% TCR$^+$	30.53 ± 3.40	13.33 ± 2.11	13.67 ± 2.12	F(2,37) = 14.57	**p < 0.001**	**p < 0.001**	**p = 0.001**	p > 0.999
# TCR$^+$ (x 10^7)	1.51 ± 0.41	0.88 ± 0.21	1.15 ± 0.43	F(2,37) = 3.21	p = 0.052			
% CD4$^+$ (TCR$^+$ pregate)	50.75 ± 2.52	45.08 ± 3.09	40.08 ± 3.09	F(2,37) = 4.78	**p = 0.014**	p = 0.163	**p = 0.014**	p = 0.655
# CD4$^+$ TCR$^+$ (x 10^6)	7.29 ± 1.88	3.68 ± 0.80	4.10 ± 1.37	F(2,37) = 4.62	**p = 0.016**	**p = 0.014**	p = 0.230	p > 0.999
% CD25$^+$ (CD4$^+$ TCR$^+$ pregate)	8.06 ± 0.32	9.75 ± 0.95	10.09 ± 1.38	F(2,37) = 1.58	p = 0.221			
# CD25$^+$ CD4$^+$ TCR$^+$ (x 10^5)	6.25 ± 1.58	3.20 ± 0.67	4.08 ± 1.38	F(2,37) = 6.39	**p = 0.004**	**p = 0.003**	p = 0.272	p = 0.434

Table S1 continued

Parameter	mean ± SEM			ANCOVA		Bonferroni posthoc		
	C	S	R			C vs. S	C vs. R	S vs. R
% $FoxP3^+$ ($CD4^+$ TCR^+ pregate)	1.17 ± 0.11	0.98 ± 0.19	1.32 ± 0.34	F(2,37) = 0.45	p = 0.643			
# $FoxP3^+$ $CD4^+$ TCR^+ (x 10^4)	7.86 ± 2.26	3.05 ± 0.75	3.22 ± 1.05	F(2,37) = 5.56	**p = 0.008**	**p = 0.009**	p = 0.070	p = 0.655
