# Supplementary material for: Social Defeat Modulates T Helper Cell Percentages in Stress Susceptible and Resilient Mice
Source: Int J Mol Sci. 2019 Jul 17;20(14):3512. doi: 10.3390/ijms20143512 (PMC6678569; doi:10.3390/ijms20143512)
Supplement: Supplementary file 1 [file ijms-20-03512-s001.zip › Supplementary_Table_2.pdf]

Table S2. Fold change of 84 genes in the mouse cytokine and chemokine RT^2 profiler array normalized to the mean expression of housekeeping genes *Gapdh*, *Gusb* and *Hsp90ab1*.

**Refseq**	**Symbol**	**Description**	**Susceptible vs. control fold regulation**	**p-value**[B]	**Resilient vs. control fold regulation**
NM_009605	*Adipoq*	Adiponectin, C1Q and collagen domain containing	-2,6964	0.220	-7.0969
NM_007553	*Bmp2*	Bone morphogenetic protein 2	-2,1802	0.149	-2.9385
NM_007554	*Bmp4*	Bone morphogenetic protein 4	-2,4283	0.111	-1.5382
NM_007556	*Bmp6*	Bone morphogenetic protein 6	-1,9336	0.093	-1.779
NM_007557	*Bmp7*	Bone morphogenetic protein 7	-2,4139	0.060	-1.8698
NM_011329	*Ccl1*	Chemokine (C-C motif) ligand 1	-2,2851	0.241	-1.2779
NM_011330	*Ccl11*	Chemokine (C-C motif) ligand 11	5,6915	0.374	1.147
NM_011331	*Ccl12*	Chemokine (C-C motif) ligand 12	-2,7573	0.092	-2.4954
NM_011332	*Ccl17*	Chemokine (C-C motif) ligand 17	-2,3891	0.127	-1.2329
NM_011888	*Ccl19*	Chemokine (C-C motif) ligand 19	-2,7219	0.091	-1.0386
NM_011333	*Ccl2*	Chemokine (C-C motif) ligand 2	-3,0426	0.302	-4.7352
NM_016960	*Ccl20*	Chemokine (C-C motif) ligand 20	-1,0439 [A]	0.895	1.6497 [A]
NM_009137	*Ccl22*	Chemokine (C-C motif) ligand 22	**-3,3055**	**0.013**	-2.6771
NM_019577	*Ccl24*	Chemokine (C-C motif) ligand 24	-1,5391	0.224	-1.2805
NM_011337	*Ccl3*	Chemokine (C-C motif) ligand 3	**-2,0926**	**0.034**	-2.5615
NM_013652	*Ccl4*	Chemokine (C-C motif) ligand 4	-1,6811	0.094	-2.1329
NM_013653	*Ccl5*	Chemokine (C-C motif) ligand 5	**-2,4572**	**0.024**	-2.1169
NM_013654	*Ccl7*	Chemokine (C-C motif) ligand 7	1,3437	0.989	1.3493
NM_011616	*Cd40lg*	CD40 ligand	-2,1442	0.053	-1.7667
NM_011617	*Cd70*	CD70 antigen	**-7,0455**	**0.019**	-1.8189
NM_170786	*Cntf*	Ciliary neurotrophic factor	-1,2059	0.841	2.0007
NM_007778	*Csf1*	Colony stimulating factor 1 (macrophage)	-2,5178	0.261	-1.3083
NM_009969	*Csf2*	Colony stimulating factor 2 (granulocyte-macrophage)	-2,2843	0.230	-1.3264
NM_009971	*Csf3*	Colony stimulating factor 3 (granulocyte)	2,5967	0.099	3.9025
NM_007795	*Ctf1*	Cardiotrophin 1	1,0819	0.790	1.6978
NM_009142	*Cx3cl1*	Chemokine (C-X3-C motif) ligand 1	-1,812	0.553	-1.0778
NM_008176	*Cxcl1*	Chemokine (C-X-C motif) ligand 1	-1,5033	0.068	-1.7245
NM_021274	*Cxcl10*	Chemokine (C-X-C motif) ligand 10	-2,58	0.052	-1.9927
NM_019494	*Cxcl11*	Chemokine (C-X-C motif) ligand 11	**-4,8526**	**0.003**	-1.5883
NM_021704	*Cxcl12*	Chemokine (C-X-C motif) ligand 12	-1,829	0.082	-1.5104
NM_018866	*Cxcl13*	Chemokine (C-X-C motif) ligand 13	-2,4888	0.075	-1.6641
NM_023158	*Cxcl16*	Chemokine (C-X-C motif) ligand 16	-3,7689	0.069	-1.7893
NM_203320	*Cxcl3*	Chemokine (C-X-C motif) ligand 3	1,6266	0.903	-1.0923
NM_009141	*Cxcl5*	Chemokine (C-X-C motif) ligand 5	1,285	0.717	-1.0211
NM_008599	*Cxcl9*	Chemokine (C-X-C motif) ligand 9	-1,4589	0.264	-1.3553
NM_010177	*Fasl*	Fas ligand (TNF superfamily, member 6)	1,0862	0.958	1.4083
NM_008155	*Gpi1*	Glucose phosphate isomerase 1	-1,5124	0.129	-1.5801
NM_010406	*Hc*	Hemolytic complement	-4,3546	0.090	-2.5876

Table S2 continued

Refseq	Symbol	Description	Susceptible vs. control fold regulation	p-value[B]	Resilient vs. control fold regulation
NM_010503	*Ifna2*	Interferon alpha 2	-1,0439 [A]	0.895	1.6497 [A]
NM_008337	*Ifng*	Interferon gamma	-1,9443	0.078	-3.2882
NM_010548	*Il10*	Interleukin 10	-1,3453	0.390	-4.4725
NM_008350	*Il11*	Interleukin 11	-1,9218	0.173	-1.6709
NM_008351	*Il12a*	Interleukin 12A	**-2,7967**	**0.014**	-2.5416
NM_008352	*Il12b*	Interleukin 12B	-2,0722	0.064	-2.4733
NM_008355	*Il13*	Interleukin 13	-1,5366	0.190	-2.3091
NM_008357	*Il15*	Interleukin 15	-1,921	0.053	-2.037
NM_010551	*Il16*	Interleukin 16	-1,9137	0.291	-2.3293
NM_010552	*Il17a*	Interleukin 17A	-1,0439 [A]	0.895	1.6497 [A]
NM_145856	*Il17f*	Interleukin 17F	1,923	0.231	3.9549
NM_008360	*Il18*	Interleukin 18	**-1,5194**	**0.026**	-1.7192
NM_010554	*Il1a*	Interleukin 1 alpha	-1,2444	0.755	-1.1079
NM_008361	*Il1b*	Interleukin 1 beta	1,1195	0.980	-1.543
NM_031167	*Il1rn*	Interleukin 1 receptor antagonist	-1,4635	0.180	-1.9358
NM_008366	*Il2*	Interleukin 2	-1,4114	0.397	-1.2369
NM_021782	*Il21*	Interleukin 21	-1,2495	0.782	-2.5174
NM_016971	*Il22*	Interleukin 22	1,1468	0.567	2.1213
NM_031252	*Il23a*	Interleukin 23, alpha subunit p19	1,0946	0.857	1.2384
NM_053095	*Il24*	Interleukin 24	-3,5384	0.124	-2.3583
NM_145636	*Il27*	Interleukin 27	**-2,972**	**0.005**	-2.3616
NM_010556	*Il3*	Interleukin 3	-1,4952	0.835	2.3884
NM_021283	*Il4*	Interleukin 4	-2,0652	0.114	-1.4104
NM_010558	*Il5*	Interleukin 5	-4,2424	0.053	-1.3287
NM_031168	*Il6*	Interleukin 6	1,3978	0.415	-1.6182
NM_008371	*Il7*	Interleukin 7	**-2,8708**	**0.001**	-1.8265
NM_008373	*Il9*	Interleukin 9	-1,0439 [A]	0.895	1.6497 [A]
NM_008501	*Lif*	Leukemia inhibitory factor	**-1,6974**	**0.007**	-1.7968
NM_010735	*Lta*	Lymphotoxin A	**-3,861**	**0.019**	-2.0008
NM_008518	*Ltb*	Lymphotoxin B	**-2,9926**	**0.018**	-1.9319
NM_010798	*Mif*	Macrophage migration inhibitory factor	1,3147	0.338	-2.603
NM_010834	*Mstn*	Myostatin	-1,0439 [A]	0.895	1.6497 [A]
NM_013611	*Nodal*	Nodal	-1,2466	0.634	1.1199
NM_001013365	*Osm*	Oncostatin M	-1,067	0.837	-1.9452
NM_019932	*Pf4*	Platelet factor 4	1,0582	0.740	-1.0346
NM_023785	*Ppbp*	Pro-platelet basic protein	1,0016	0.770	1.0861
NM_009263	*Spp1*	Secreted phosphoprotein 1	**-1,6097**	**0.023**	-1.8453
NM_009367	*Tgfb2*	Transforming growth factor, beta 2	-1,378	0.144	1.0267
NM_009379	*Thpo*	Thrombopoietin	-1,1805	0.700	-1.8442
NM_013693	*Tnf*	Tumor necrosis factor	**-3,5215**	**0.010**	-2.2974
NM_008764	*Tnfrsf11b*	Tumor necrosis factor receptor superfamily, member 11b (osteoprotegerin)	**-3,5001**	**0.035**	-1.3698

Table S2 continued

Refseq	Symbol	Description	Susceptible vs. control fold regulation	p-value[B]	Resilient vs. control fold regulation
NM_009425	*Tnfsf10*	Tumor necrosis factor (ligand) superfamily, member 10	-1,6369	0.349	1.0805
NM_011613	*Tnfsf11*	Tumor necrosis factor (ligand) superfamily, member 11	-2,0835	0.243	-2.6957
NM_033622	*Tnfsf13b*	Tumor necrosis factor (ligand) superfamily, member 13b	-1,8146	0.263	1.2428
NM_009505	*Vegfa*	Vascular endothelial growth factor A	**-2,0575**	**0.002**	-6.3409
NM_008510	*Xcl1*	Chemokine (C motif) ligand 1	**-2,1714**	**0.008**	-2.5541

A: This gene’s average threshold cycle is either not determined or greater than the defined cut-off (35 Ct), in both samples meaning that its expression was undetected, making this fold-change result erroneous and uninterpretable.

B: p-value from t-test between control (n = 3) and susceptible (n = 3) samples. For resilient mice (n = 2) statistics could not be calculated due to low sample size.
