# Supplementary material for: Social Defeat Modulates T Helper Cell Percentages in Stress Susceptible and Resilient Mice
Source: Int J Mol Sci. 2019 Jul 17;20(14):3512. doi: 10.3390/ijms20143512 (PMC6678569; doi:10.3390/ijms20143512)
Supplement: Supplementary file 1 [file ijms-20-03512-s001.zip › Supplementary_Table_3.pdf]

Table S3. Overview of cohorts and sample sizes for flow cytometry experiments. C: control, S: susceptible, R: resilient.

Spleen cells unstimulated*	Sample size behavior			Parameters flow cytometry	Sample size flow cytometry		
	C	S	R		C	S	R
Cohort 1	5	5	2		-[a]	-[a]	-[a]
Cohort 2	5	6	4	TCR, CD4, CD8, IFNg, IL-17A	5	6	4
Cohort 3	5	5	5	TCR, CD4, CD8, IFNg, IL-17A	5	5	5
Total	15	16	11		10	11	9

PMA/Iono stimulated spleen cells*	Sample size behavior			Parameters flow cytometry	Sample size flow cytometry		
	C	S	R		C	S	R
Cohort 1	5	5	2		-[b]	-[b]	-[b]
Cohort 2	5	6	4	TCR, CD4, CD8, IFNg, IL-17A	4[c]	6	4
Cohort 3	5	5	5	TCR, CD4, CD8, IFNg, IL-17A	5	5	5
Total	26	26	21		9	11	9

Intranuclear FoxP3 stained spleen cells*	Sample size behavior			Parameters flow cytometry	Sample size flow cytometry		
	C	S	R		C	S	R
Cohort 1	5	5	2	TCR, CD4, CD25, FoxP3	5	5	2
Cohort 2	5	6	4	TCR, CD4, CD25, FoxP3	5	6	4
Cohort 3	5	5	5	TCR, CD4, CD25, FoxP3	5	5	5
Total	15	16	11		15	16	11

* Same animals were used for the indicated flow cytometric analyses. [a] Unstimulated spleen cells were not investigated in this cohort. [b] PMA/Iono stimulated spleen cells were not investigated in this cohort. [c] One sample had not been stimulated with PMA/Iono.
